# Sociological analysis of nurses’ qualification through stricto sensu graduate programs globally: scoping review

**DOI:** 10.1590/0034-7167-2024-0560

**Published:** 2026-07-10

**Authors:** Bárbara Costa Moreira, Isabella Lara Maia de Carvalho, Rafaela Siqueira Costa Schreck, Camila Pureza Guimarães da Silva, Maria Itayra Coelho de Souza Padilha, Fernanda Batista Oliveira Santos

**Affiliations:** I Universidade Federal de Minas Gerais. Belo Horizonte, Minas Gerais, Brazil; II Universidade Federal do Rio de Janeiro. Rio de Janeiro, Rio de Janeiro, Brazil; III Universidade Federal de Santa Catarina. Florianópolis, Santa Catarina, Brazil

**Keywords:** Nursing, History of Nursing, Education, Nursing, Graduate, Education, Nursing, Nursing Education Research., Enfermería, Historia de la Enfermería, Educación de Postgrado en Enfermería, Educación en Enfermería, Investigación en Educación de Enfermería.

## Abstract

**Objectives:**

to analyze the landscape of scientific publications regarding the historical process of nurses’ qualification through *stricto sensu* graduate programs in Nursing on a global scale, in light of Eliot Freidson’s Sociology of Professions.

**Methods:**

a scoping review encompassing the databases BVS, MEDLINE, Embase, Cochrane Library, Scopus, and Web of Science. The descriptors used were “History of Nursing,” “History and Graduate Education in Nursing,” and their equivalents in English and Spanish, following the recommendations of the Joanna Briggs Institute and the PRISMA Extension for Scoping Reviews guideline. The final sample included twenty articles.

**Results:**

*stricto sensu* graduate education in Nursing has been consolidated differently across countries, influenced by educational policies, international cooperation, and professional strengthening strategies.

**Final Considerations:**

the consolidation of graduate education in Nursing has shown global variations, reinforcing professional autonomy and the development of knowledge. This study contributes to the historical understanding of Nursing professionalization.

## RESUMEN

**Objetivos:**

analizar el panorama de las publicaciones científicas sobre el proceso histórico de la cualificación de los enfermeros a través de los programas de posgrado *stricto sensu* en Enfermería a nivel global, a la luz de la Sociología de las Profesiones de Eliot Freidson.

**Métodos:**

revisión de alcance, abarcando las bases de datos BVS, MEDLINE, Embase, Cochrane Library, Scopus y Web of Science. Se utilizaron los descriptores “Historia de la Enfermería”, “Historia y Educación de Posgrado en Enfermería” y sus equivalentes en inglés y portugués, siguiendo las recomendaciones del *Joanna Briggs Institute* y la directriz *PRISMA Extension for Scoping Reviews*. La muestra final incluyó veinte artículos.

**Resultados:**

la educación de posgrado *stricto sensu* en Enfermería se consolidó de manera distinta en los países, influenciada por políticas educativas, cooperación internacional y estrategias de fortalecimiento profesional.

**Consideraciones Finales:**

la consolidación de la educación de posgrado en Enfermería presentó variaciones globales, fortaleciendo la autonomía profesional y la construcción del conocimiento. El estudio contribuye a la comprensión histórica de la profesionalización de la Enfermería.

## INTRODUCTION

The evolution of nursing as a profession is closely linked to the institutionalization of education, particularly through *Stricto Sensu* Graduate Programs in Nursing (*“PPGENF”*). These programs, as mechanisms of professionalization shaped by social and public health transformations, emphasize the pursuit of autonomy and recognition of nurses’ expertise-a dynamic interpreted through Eliot Freidson’s Sociology of Professions^(1,2)^.

Sociologist Freidson argues that for an occupation to be recognized as a profession, it must possess essential elements: its own educational institutions, a credentialing system, and specific regulation of practice and autonomy. Furthermore, the profession must be grounded in deep and hierarchical intellectual knowledge, which requires a high level of professional qualification^(1)^.

Historically, modern nursing began to consolidate in England during the second half of the 19th century with Florence Nightingale, who established the need for formal and standardized training for nurses. The Nightingale model was later adapted and disseminated globally, especially by the United States of America (USA), incorporating capitalist and Taylorist characteristics into nursing education. This adaptation aimed to increase efficiency in training professionals in the field^(3,4)^.

The institutionalization of Graduate Nursing Education in the United States guided the practices of other countries, as it was the first nation to offer master’s and doctoral programs^(5-8)^. Since 1924, Teacher’s College, located in Columbia, Ohio, had already been offering doctoral programs, with its graduates earning the title of Doctor of Education (PhD). This degree enabled nurses to work in teaching roles, although it was not a program specifically designed for nursing or health sciences. The first doctoral program specifically related to nursing was established in 1934 in New York, marking a significant milestone in the professionalization process^(9)^.

The expansion of *stricto sensu* graduate education in Nursing was not limited to the American context. Several countries, especially in Latin America, began investing in the academic qualification of nurses, promoting the development of specialized knowledge and strengthening the profession^(4)^. In this context, Brazil assumed a prominent role, leading initiatives in the training of master’s and doctoral graduates and contributing significantly to the consolidation of Nursing as both a scientific and professional field in the region^(4,5)^.

## OBJECTIVES

To analyze the landscape of scientific publications on the historical process of nurses’ qualification through *stricto sensu* graduate programs in nursing on a global scale, in light of Eliot Freidson’s Sociology of Professions.

## METHODS

### Type of Study

This study is a scoping review aimed at identifying the types of evidence available within a specific field of study, analyzing knowledge gaps, and identifying the main characteristics or factors related to a given concept^(10)^. In this context, the following research question was formulated: “On a global scale, how did the historical process of nurses’ qualification unfold through the institutionalization of *stricto sensu* graduate education in Nursing?”.

For this scoping review, the recommendations of the Joanna Briggs Institute (JBI) were followed, based on the methodological framework developed by Arksey and O’Malley, and structured using the PRISMA-ScR (Preferred Reporting Items for Systematic Reviews and Meta-Analyses extension for Scoping Reviews) guideline. The review was conducted by two evaluators and supported by a publicly registered research protocol on the Open Science Framework platform (https://osf.io/vm4bd/)^(11,12)^.

### Methodological Procedures

To conduct this study, five steps were followed: 1) Formulation of the guiding research question; 2) Definition of the research objectives, establishing inclusion and exclusion criteria; 3) Development of the search strategy and selection of Health Sciences Descriptors (MeSH); 4) Categorization of the studies found; 5) Interpretation and presentation of the results.

### Data Collection and Organization

Data collection was conducted from January to March 2024 through the LILACS database and other sources via the Regional Portal of BVS, MEDLINE via PubMed, and, through the CAPES portal, Embase, Cochrane Library, Scopus, and Web of Science. Studies were retrieved that contained at least one of the terms related to the concepts of History of Nursing and Graduate Education in Nursing. Controlled vocabulary terms (MeSH) and free terms (keywords) were identified, including History of Nursing and History and Graduate Education in Nursing, along with their equivalents in English and Spanish.

### Sources Selection Criteria

The texts retrieved were selected based on an independent evaluation of titles and abstracts conducted by a primary and a secondary reviewer using Rayyan^®^ software. This study included scientific articles, editorials, and research notes in all languages. No time limit was set for inclusion, given the need to map the literature in a broad and comprehensive manner. Texts that were not available in full or did not address the topic of *Stricto Sensu* Graduate Education were excluded. Eligibility was determined by the presence of content related to *Stricto Sensu* Graduate Education in the global context.

The texts were pre-selected through independent title and abstract screening by a primary and a secondary reviewer. Any disagreements regarding selection were resolved through consensus meetings between reviewers. The pre-selected articles were read in full to assess their relevance to the study and to more precisely apply the inclusion and exclusion criteria. In this final stage, the most relevant data were extracted for analysis.

### Data Analysis

The analysis of the studies was conducted descriptively, grouping the articles according to the following aspects: socio-historical contexts, institutional arrangements, and governmental incentives for the institutionalization of *stricto sensu* graduate programs worldwide. Subsequently, a narrative synthesis was carried out, highlighting the methodological and thematic characteristics identified in the studies.

## RESULTS

**Figure 1:**
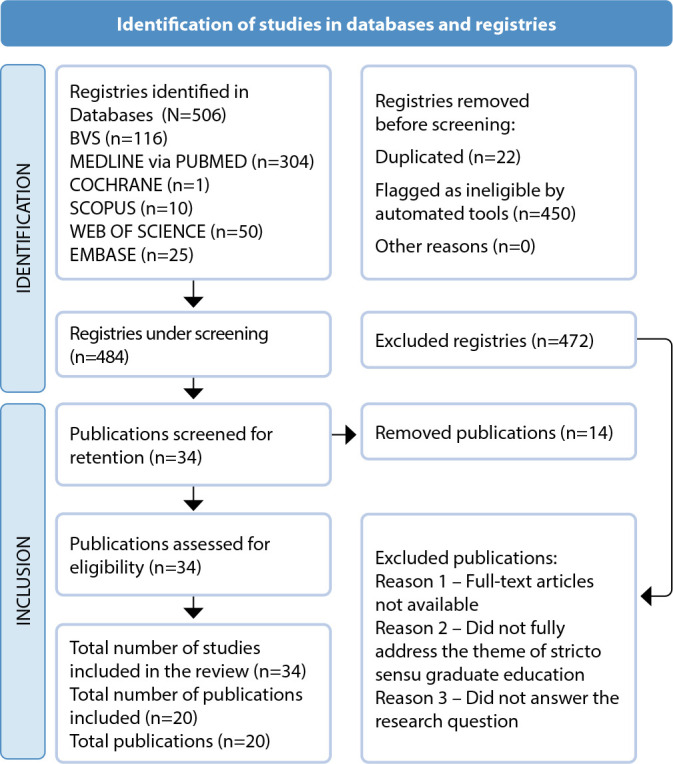
Diagram of the article search and selection process, according to an adaptation of PRISMA-Scr

The final sample of this scoping review comprised twenty selected studies, classified as levels of evidence V and VI^(13)^. The process of searching and including articles is illustrated in the flowchart (Figure 1), following the recommendations of the JBI, based on an adapted checklist from the Preferred Reporting Items for Systematic Reviews and Meta-Analyses Extension for Scoping Reviews (PRISMA-ScR)^(10-12)^.

**Figure 2:**
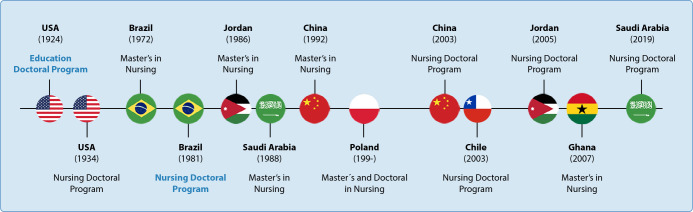
Timeline of the emergence of *stricto sensu* graduate education in Nursing worldwide

The studies addressed the historical process of nurses’ qualification through *stricto sensu* graduate education in Brazil (n=13), Chile (n=1), China (n=2), Poland (n=1), Saudi Arabia (n=2), Jordan and Ghana (n=1), and the United States of America (n=1). Considering the emergence of *stricto sensu* graduate programs in Nursing within the global context of this sample, Figure 2 outlines the chronological beginning of master’s and/or doctoral programs.

The characterization of the scientific production regarding the historical process of qualification through *stricto sensu* graduate education in the global context is presented in (Chart 1).

**Chart 1:** Characterization of the scientific production regarding the historical process of qualification through stricto sensu graduate education in the global context

Code ID	Country / Year	Title	Delineation	Level of Evidence	Interventions and Outcome
PC1^(14)^	Brazil (2002)	*The graduate programs offered by the University of São Paulo at Ribeirão Preto college of nursing: historical evolution and contributions to nursing development*	Quantitative analysis of graduate-level academic production and bibliographic sources on the subject.	Level V	The *Escola de Enfermagem de Ribeirão Preto da Universidade de São Paulo* (*“EERP-USP”*, Ribeirão Preto School of Nursing at the University of São Paulo) has contributed significantly to the training of researchers and was designated in 1988 as a Collaborating Center of the World Health Organization for the development of nursing research. With its consolidated Graduate Program, EERP became a reference in establishing partnerships with other institutions for doctoral training. Institutional agreements were signed with the *Universidade Federal da Bahia (“UFBA”*, Federal University of Bahia) (*“UFBA”*, March 1995), the *Universidade Federal da Paraíba* (“UFPA, Federal University of Paraíba (*“UFPA”,* March 1995), the University of Concepción - Chile (UdeC, March 1996), and the Universidade Federal do Rio Grande do Norte (*“UFRN”*, Federal University of Rio Grande do Norte) (*“UFRN”*, March 1997).
PC2^(7)^	Chile (2004)	*Stricto Sensu graduate program at the Nursing Department of Concepción University: Concepción, Chile*	Documentary Analysis.	Level V	The Nursing program at the University of Concepción was established in 1947. Due to significant epidemiological changes in the country during the 2000s, the need to implement *stricto sensu* graduate programs became evident. This study does not document the establishment of the university’s Master’s program in Nursing. However, the Doctoral program in Nursing began its academic activities in March 2004, supported by the *Universidade de São Paulo*.
PC3^(15)^	Brazil (2005)	*The history of the nursing interunits doctoral program and its contribution to Brazilian nursing*	Documentary Analysis.	Level V	The Schools of Nursing at the *University of São Paulo* (*“USP”*), located on the São Paulo and Ribeirão Preto campuses, shared the aspiration to create a doctoral program in the field. In 1979, both Schools submitted proposals to *“USP”*’s Graduate Studies Council for the establishment of the program. The final assessment concluded that there was not yet a sufficient critical mass for each unit to offer the doctorate independently, recommending instead the organization of a joint program involving both schools. This initiative was carried out by professors Wanda de Aguiar Horta, Amália Correia de Carvalho, Evalda Cançado Arantes, Maria Aparecida Minzoni, Maria Helena Machado, and Nilza Tereza Rotter Pelá. The Inter-Unit Doctoral Program in Nursing was approved by *“USP”*’s Graduate Studies Council in 1981, marking the establishment of the first Nursing Doctorate in Brazil and Latin America. In June 1986, the program was officially accredited by the *Conselho Federal de Educação* (*“CFE”*, Federal Council of Education), now known as the Conselho Nacional de Educação (*“CNE”*, National Council of Education).
PC4^(16)^	Brazil (2005)	*The University of São Paulo, School of Nursing’s history of graduate program*	Historical and qualitative study.	Level VI	The need for Graduate Education in Nursing was legally recognized in 1949, as Decree No. 27,426, which regulated Law No. 775 of 1949 concerning nursing education in Brazil, stated in Article 3 that “in addition to the two regular courses, other graduate-level programs may (or could) be created to expand specialized knowledge in nursing or administration.”.
PC5^(9)^	United States of America (2005)	PhD, DNSc, ND: the ABCs of nursing doctoral degrees	Historical and qualitative study.	Level VI	Doctoral education in the United States has its roots in Europe, being strongly influenced by the German educational model. The first doctoral degree in the U.S. was awarded in 1924 at Teachers College, Columbia University (CU). In 1934, New York University (NYU) offered the first Doctorate in Nursing. In 1950, the University of Pittsburgh launched the first Nursing Doctorate with an emphasis on nursing care delivery. By the 1960s, four types of doctoral degrees existed: EdD (Doctor of Education); DNSC (Doctor of Nursing Science); ND (Doctor of Nursing), a clinically oriented graduate degree without a research focus; and a generic doctoral program.
PC6^(17)^	China (2005)	International collaboration for developing graduate education in China	Historical and qualitative study.	Level V	In 1990, the Committee of Graduate Nursing Education (COGNE) sent fifteen Chinese nursing professionals to the United States for advanced training, with the goal of updating the faculty through the completion of Master of Science in Nursing degrees. Of those fifteen professionals, only four returned to China after the opportunity. In 1994, the Higher Education Bachelor’s Program (POHNED) was launched at Xi’an Medical University (XMU), marking the first graduate-level nursing program in China. Between 1994 and 2001, this program trained 84 nurses at the master’s level.
PC7^(18)^	Brazil (2006)	*A success story: 30 years of the Nursing Graduate Program at UFSC*	Socio-historical and qualitative research.	Level VI	During the 1950s and early 1960s, there was a strong emphasis on training higher education professionals abroad, supported by funding agencies such as the *Conselho Nacional de Desenvolvimento Científico e Tecnológico* (*“CNPq”*, National Council for Scientific and Technological Development), the *Coordenação de Aperfeiçoamento de Pessoal de Nível Superior* (*“CAPES”,* Coordination for the Improvement of Higher Education Personnel), the *Fundo do Desenvolvimento Técnico e Científico* (*“FNDCT”,* Fund for Technical and Scientific Development), the *Fundação de Amparo à Pesquisa do Estado de São Paulo* (*“FAPESP”,* São Paulo Research Foundation), and international institutions such as the Rockefeller Foundation and the Ford Foundation. Several factors contributed to the creation of graduate programs in Brazil, including the mobilization for university reform, the growth in higher education enrollment, the quantitative expansion of institutes and departments, the need for more qualified faculty, and the decisive increase in demand for graduate education.
PC8^(19)^	Brazil (2010)	*Contribution of Anna Nery Nursing School for master´s education in nursing in Brazil (1972-1982)*	Socio-historical and qualitative research.	Level V	The Brazilian government sought international assistance by establishing agreements between the Ministry of Education and Culture (MEC) and the United States Agency for International Development (USAID), aimed at reforming higher education. One of the main points agreed upon was that educational reform should align with the country’s economic expansion needs. In the late 1960s, the government created commissions to draft proposals for the reform of Brazilian education. At the end of 1967, the Meira Mattos Commission was established and presented proposals with immediate impact. In 1968, the government created the University Reform Working Group (*“GTRU”*), which proposed broader reforms for higher education, based on the MEC/USAID agreements. Nursing leadership in Brazil had already recognized the importance of scientific research for the development of the profession and the construction of its body of knowledge. A landmark event reflecting this concern was the 16th *Congresso Brasileiro de Enfermagem* (Brazilian Nursing Congress), organized by the *Associação Brasileira de Enfermagem* (“*ABEn”* Brazilian Nursing Association).
PC9^(20)^	Brazil (2010)	*The development of post-graduate programs in nursing in Brazil: an exploratory study*	Documentary research.	Level VI	One of the earliest strategies for qualifying nursing professionals to teach in *stricto sensu* graduate programs was the implementation of public examinations for “*livre-docência”* and doctoral degrees, which conferred academic titles upon those approved. Candidates were required to possess a researcher’s *habitus*, as they had to defend a thesis or present a previously completed scholarly work to participate in the selection process. At the *Escola de Enfermagem Anna Nery (EEAN)*, doctoral and “*livre-docência”* examinations took place in 1968, 1975, and 1977. Consequently, from the 1970s onward, Master’s and Doctoral programs in Nursing were established.
PC10^(21)^	China (2012)	*Graduate nursing education in China*.	Historical and qualitative research.	Level V	In 1978, China adopted an Open Policy model upon recognizing the advanced state of higher nursing education in other countries. As a result, the first School of Nursing was established in 1983 at Tianjin Medical University (TMU). Master’s programs in China began in 1992 at Peking Union Medical College (PUMC), expanding to five programs by 1998, thirty by 2003, fifty-eight by 2007, and sixty-five by 2009. In China, there are two types of Master’s programs: research-oriented and clinical. The first Doctoral program was launched in 2003 at the Second Military Medical University (SMMU). One of the challenges faced by graduate students is the prevailing perception that nursing is a subdiscipline of medicine, which influences and constrains the structure of graduate education.
PC 11^(22)^	Brazil (2012)	*The post-graduation program of Brazilian Nursing completes forty years: developments, challenges and the need of new investments to improve it*	Editorial.	Level V	Graduate Nursing Programs have been training new researchers globally, a movement that has driven the expansion of Brazilian Graduate Nursing Education. Its visibility has been built through the increase in the number of programs, the consolidation of qualified human resource training, research, and scientific production. This consolidation is reflected in the 523 Research Groups registered with the *Conselho Nacional de Desenvolvimento Científico e Tecnológico (CNPq)*, the 165 *“CNPq”* Researcher Grants, the 5,194 articles published between 2007 and 2009 with a noticeable improvement in journal quality, the indexing of four Brazilian Nursing journals in the Web of Science in 2010, and, finally, the achievement of four programs recognized for their national and international excellence.
PC12^(23)^	Brazil (2013)	*Graduate program in nursing at the Federal University of Santa Maria: trajectory and results*	Quantitative, descriptive, exploratory, and documentary study.	Level V	In the 1920s, nurses who graduated from the *Escola de Enfermagem Ana Nery* began postgraduate studies in the United States through scholarship programs. As a result, the Southeast region of Brazil saw the first investments in professional development related to graduate education, serving as a driving force for the creation of similar educational spaces in other regions of the country. However, it is currently observed that resources and funding remain concentrated in certain regions, revealing asymmetries in the landscape of graduate nursing education in Brazil.
PC13^(24)^	Brazil (2013)	*The Strict Sense Nursing postgraduation in Brazil: advances and perspectives*	Socio-historical and qualitative study.	Level V	Brazilian graduate education was established by the *Lei de Diretrizes e Bases da Educação Nacional* (National Education Guidelines and Framework Law) in 1961 and officially approved in 1965. It emerged within a political context aimed at promoting the country’s economic development, driven by the need for specialized labor to fill new jobs created by projected growth, as well as the demand for scientists, researchers, and technical professionals. Nursing was recognized as a distinct field of knowledge with representation in the Technical-Scientific Council (CTC) of the *Coordenação de Aperfeiçoamento de Pessoal de Nível Superior (CAPES)*, through Resolution No. 1 of 1987. Faced with challenges, Nursing Schools and the field’s representatives within *“CAPES”* mobilized to find alternative strategies to enable the graduate-level training of qualified human resources. Another important milestone was the reform in the course evaluation system, marked by increased rigor in assessment criteria, a triennial evaluation cycle, and program ratings on a scale from 1 to 7.
PC14^(25)^	Brazil (2015)	*Post-graduate in nursing in the University of Brasília historical aspects of a process of collective construction*	Historical and qualitative study.	Level V	In the 1990s, the faculty of Graduate Nursing Programs in Brazil was predominantly composed of nurse-doctors from within the institution itself. When this was not the case, faculty members from other academic disciplines within the same institution were incorporated into the programs.
PC15^(26)^	Brazil (2015)	*Nursing master’s program at Anna Nery school 1972-1975: singularities of graduating and challenges in its implementation*	Socio-historical and qualitative study.	Level V	For the implementation of the first Master’s program in Nursing at the Escola de Enfermagem Anna Nery , the Ministry of Education (*“MEC”*) accredited faculty members for *stricto sensu* graduate education who had completed postgraduate studies abroad or at other Brazilian public universities. To complete the faculty composition, professors from other fields, such as education and philosophy, were invited, provided they already held a master’s degree or were accredited to teach in *stricto sensu* graduate programs.
PC16^(27)^	Poland (2018)	*Nursing education in Poland - The past and new development perspectives*.	Historical and qualitative study.	Level V	The first Master’s Program in Nursing was established in 1977 at the University of Medical Sciences in Poznań. However, the transformation of the nursing system in Poland, resulting from the country’s liberation from Soviet influence, was consolidated in 1989 with the democratization of the political system. In this context, nurses began intensive and multidimensional efforts to redesign their practice and education at both basic and advanced levels. Starting in 1995, the Department of Nurses and Midwives at the Ministry of Health in Warsaw initiated intensive work to define the functional competencies of nursing care within the framework of the new educational model. The key reference document was the “European Strategy of the World Health Organization (WHO) for the Education of Nurses and Midwives”, adopted in 1999. That same year, the Bologna Declaration was signed by 30 representative European countries, a document that helped shape the current structure of nursing education in Poland. Following its adoption, significant changes were introduced in nursing education, including the implementation of a three-tier system: Bachelor’s in Nursing, Master’s in Nursing, and Doctorate in Nursing.
PC17^(28)^	Saudi Arabia (2020)	*Nursing Education in Saudi Arabia: History and Development*	Integrative literature review.	Level V	The first Master’s Program in Nursing in the Kingdom of Saudi Arabia was established in 1987 at King Saud University (KSU), exclusively for female students. Starting in 2013, male students were allowed to enroll. The only Doctoral Program in Nursing was introduced in 2019.
PC18^(29)^	Saudi Arabia, Jordan, Ghana (2020)	*Development of nurse education in Saudi Arabia, Jordan and Ghana: From undergraduate to doctoral programs*	Integrative literature review.	Level V	The earliest records of Master’s programs in Nursing in Saudi Arabia date back to 1988, initially offered exclusively to women until 2014. The first Master’s Program in Nursing was established at King Saud University, which also launched the country’s first Doctoral Program in Nursing in 2019. In Jordan, the Master’s program began in 1986, with the entire faculty composed of foreign professionals from the United States, the United Kingdom, and Sweden. The Doctoral Program was created in 2005 at the University of Jordan (JU). In Ghana, prior to independence in 1957, the country followed the nursing curriculum established by the General Nursing Council of England and Wales. After gaining independence, Ghana formed the Registered Nursing Association to maintain a registry of nurses, and-similar to the United Kingdom-nursing education focused on curative hospital-based care. No university offered a doctoral program in nursing, although Master’s programs were available. Faculty members holding doctoral degrees were educated in Europe, the United States, South Africa, and Asia, while the remaining faculty earned doctorates in other departments of Ghanaian universities, such as public health, health administration, and psychology.
PC19^(5)^	Brazil (2021)	*UFSC graduate program in nursing: 45 years of contributions to the internationalization of brazilian nursing*	Quantitative, descriptive, exploratory, and documentary study.	Level V	Brazilian nursing established its first *stricto sensu* Graduate Program at the *Escola de Enfermagem Anna Nery* in 1972. During that same decade, eight additional Master’s programs in Nursing were created across various Brazilian states. The creation of these programs aimed to meet the growing need for qualified faculty and researchers to support higher education demands, accompany the development of *stricto sensu* graduate programs, and strengthen research through the formation of research groups and laboratories. These efforts also sought to promote the establishment of doctoral programs and, consequently, enhance international visibility.
PC20^(4)^	Brazil (2021)	*Historicity of nursing graduate studies in Brazil: an analysis of the Sociology of the Profession*	Integrative literature review.	Level VV	The institutionalization of graduate education in Brazil occurred within a context of economic development during the military regime, as a strategic effort to generate a qualified workforce and shift the country toward a research-oriented model. Considering that graduate programs in Brazil were initially designed to train professionals for teaching in higher education and to advance science and evidence-based practice, this phenomenon in Brazilian nursing began to gain momentum in the 1960s with the development of lato sensu specialization programs. It was consolidated with the approval of the first Master’s program in Nursing at the *Escola de Enfermagem Anna Nery* in 1972, and the first Doctoral program in Nursing at the School of Nursing of the *Universidade de São Paulo* in 1981.

## DISCUSSSION

This review mapped the scientific literature concerning the global historical process of nurses’ qualification through *stricto sensu* graduate education in Nursing. The articles found were classified as levels of evidence V and VI due to the retrospective and observational nature of these studies, which often rely on documentary sources, subjective accounts, and historical records. These findings enabled the researchers to interpret Freidson’s concepts in relation to the sociological dynamics of the professionalization process in nursing^(1)^.

Professions are influenced by both intrinsic and extrinsic factors, including historical, social, political, and cultural contexts^(4)^. In nursing, the professionalization process occurs through the transition from empirical care to scientific practice, resulting in a distinct body of knowledge that combines evidence-based practices with deep, systematized theoretical understanding^(30,31)^. According to Freidson’s framework, a profession differs from an occupation by exerting control over its own work, which requires mastery of three key aspects: knowledge, credentialism, and autonomy^(1)^.

In this context, the institutionalization of Graduate Education in Nursing represents a fundamental milestone in the professionalization process, as it involves the development of a specialized body of knowledge and professional control over training and practice within technical, technological, scientific, and ethical standards, requiring a systematization of care delivery^(32,33)^.

It is observed that the majority of the studies found refer to the history of the institutionalization of *Stricto Sensu* Graduate Education in Nursing in Brazil (65%)^(4,15-26)^. The significant volume of historiographic production in the sample reflects the commitment of Brazilian researchers to document, through historical analysis, the advancement of nursing professionalization in the country, an important movement for building the profession’s collective memory. However, it is worth noting that some studies adopt descriptive or analogical approaches to history, without necessarily relying on the methodological foundations of historiography. The similarity and analytical potential within the field of nursing as a profession have also been discussed in Brazil by scholars of nursing history^(32,34-36)^.

The implementation of graduate education in Brazil occurred during a period of significant political and economic change^(5)^. Under a military regime, the country experienced a transformative context driven by a combination of economic, industrial, and technological policies that promoted substantial economic growth. Industrial advancement created the need for a more qualified professional workforce, leading to the rise of student movements demanding greater access to education^(4,5,25)^. Within this context, the University Reform of 1968 was enacted, aiming to improve university conditions, strengthen faculty training, establish academic research, and enhance the visibility of higher education programs in Brazil^(4,26)^.

In 1972, the first Master’s program in Nursing in Brazil was established at the *Escola de Enfermagem Anna Nery* (*“EEAN”*, Anna Nery School of Nursing), the country’s first nursing school modeled after the Nightingale standard^(26)^. This educational model, created by Florence Nightingale in 1860, became the benchmark for subsequent nursing schools, introducing a methodical teaching practice. Nursing thus transitioned from an empirical activity, disconnected from specialized knowledge, to a disciplinary and scientific endeavor, becoming an institutionalized social practice specifically designed to meet hospital needs^(37)^.

The first Doctoral program in Nursing in Brazil and Latin America was created in 1981 at the *Escola de Enfermagem da Universidade de São Paulo* (*“EEUSP”*, School of Nursing of the University of São Paulo)^(15)^. This milestone highlights the leadership and pioneering role of Brazilian nursing in consolidating a profession grounded in the production of esoteric knowledge. Freidson explains that esoteric knowledge, referred to as expertise, is an essential component of a profession’s technical and theoretical authority, as it underpins autonomy through the development of specialized knowledge within educational institutions^(1)^.

Globally, the United States of America (USA) played a crucial role in the professionalization of nursing. Several studies identify the country as a key reference and influential supporter in advancing nursing professionalization worldwide^(4,17,19,29)^. The Anglo-American model, based on the principles of modern nursing and internationally disseminated by the USA, represents an evolution of nursing practice that emphasizes autonomy and the independent role of the nurse, and has served as a major influence in the professionalization of nursing scientific production^(38)^.

Carregal notes that while American nursing was already offering *stricto sensu* and lato sensu specialization programs in 1924, Brazil was still in the process of establishing its first nursing schools based on the Nightingale paradigm^(4)^. Moreover, the United States of America contributed to the qualification of Brazilian faculty, facilitating not only the establishment of higher education in nursing within the country but also the development of graduate programs^(38)^.

China was among the countries influenced by the USA in the professionalization of nursing^(17)^. The first nursing school was established in 1920, but within a context marked by strict state control, with an emphasis on ideological uniformity over professional and educational development. During this period, nursing was not considered an essential profession in the country, leading to the closure of several training centers. Due to socioeconomic and political factors, and with the adoption of an open government in 1978, state leaders recognized the importance of developing higher education in nursing to keep pace with global progress. In 1983, the Tianjin Medical University School of Nursing (TJMES) was founded^(21)^.

To meet the demand for qualified professionals, China established international agreements and collaborations to enhance nursing education. From a Freidsonian perspective, these efforts support the recognition of nursing as an autonomous profession, since autonomy stems from a specialized body of knowledge acquired through credentials established by the institutionalization of schools and universities^(2)^. Foreign support introduced international standards of nursing education and practice in the country, and the government created the Committee of Graduate Nursing Education (COGNE) to send Chinese nurses to the United States for faculty development. Of the fifteen professionals selected, only four returned to China after the opportunity^(17)^.

With the immigration of human capital, continuous growth of the profession, and increasing demand for skilled labor, the first Master’s program in Nursing in China was established in 1992 at the Peking Union Medical College (PUMC). The doctoral program was later instituted in 2003 at the Second Military Medical University (SMMU)^(21)^. Thus, nursing in China began to take shape as both a science and a profession through the development of its own field of knowledge to support professional practice.

As in other countries, Chile underwent economic and social reconfigurations, resulting in educational and health reforms aimed at improving the quality of healthcare. Within this sample, the emergence of the first Master’s program in Nursing in Chile is not documented. However, studies indicate that, under the influence of other countries, an initiative was launched to build a critical mass of doctoral-level academics with the goal of establishing the first Doctoral Program in Nursing in Chile, at the University of Concepción (*“UdeC”*)^(7)^.

This program was strongly influenced by the *Universidade de São Paulo Campus Ribeirão Preto* (University of São Paulo, Ribeirão Preto campus), which established a partnership with the University of Concepción, funded by the Kellogg Foundation, a U.S.-based organization. In March 1996, four Chilean students were sent to the *Universidade de São Paulo* (*“USP”*, University of São Paulo) to participate in the program. After completing their doctorates, they returned to Chile and assumed important academic positions. With these credentials, they led the creation of the first Doctoral Program in Nursing in Chile, which was officially approved in 2003^(7)^.

African and Middle Eastern countries such as Saudi Arabia, Ghana, and Jordan were also influenced by the United States. Graduate education in nursing in Saudi Arabia emerged within a context of educational development and modernization of the country’s healthcare system, which underwent significant socioeconomic transformations following the discovery of oil in the 1930s. Initially, the government focused on importing foreign knowledge and training nurses at a basic level. However, driven by the need to prepare professionals capable of addressing the shortcomings of the Saudi healthcare system, the first Master’s program in Nursing was established in 1988 at King Saud University (KSU), and was offered exclusively to women until 2014. The Doctoral program in Nursing was later introduced in 2019 at the same university^(28,29)^.

As in other countries included in this review, the Jordanian healthcare system was influenced by the Anglo-American model as the country sought to modernize its nursing practices and enhance the competence of its healthcare professionals. Jordanian universities, in collaboration with international institutions, established graduate programs in Nursing that incorporated international standards. In Jordan, the Master’s program began in 1986, with the entire faculty composed of foreign professionals from the United States, the United Kingdom, and Sweden. The doctoral program was instituted in 2005 at the University of Jordan (JU)^(29)^.

Historically, as in other African countries, Ghana has faced considerable challenges in terms of healthcare infrastructure, qualified human resources, and access to adequate healthcare services. The need to improve the conditions of the developing healthcare system prompted the government to collaborate with international institutions to establish graduate programs. The first Master’s program was instituted in 2007 at the School of Nursing and Health Sciences at the University of Ghana. Initially, the faculty was trained in Europe, the United States, South Africa, and Asia. There are no studies in this sample regarding doctoral programs in Nursing in Ghana; however, some nurses have earned doctorates in other departments of Ghanaian universities, such as public health, health administration, and psychology^(29)^.

After World War II, Europe underwent significant social, economic, and cultural changes that impacted the health of European society. Poland, an Eastern European country that was militarily occupied by Germany, experienced numerous epidemics in the post-war period, including outbreaks of infectious diseases, as well as the dismantling of pre-war achievements in Polish culture, science, and education^(27)^.

During this post-war period, due to the sociopolitical context and the urgent need for nursing professionals, Poland relied on workers with limited training, often completing six-month secondary-level courses to meet hospital demands. At the same time, some pre-war nursing schools were reestablished, along with the Polish Nursing Association, which was responsible for founding the Faculty of Nursing at the Medical Academy of Lublin in 1969, offering a three-year program. This initiative led to the creation of other nursing schools, including those in Poznań, Kraków, Katowice, and Wrocław, where nurses were trained at the graduate level to earn the title of Master in Nursing. The first Master’s program in Nursing was established in 1977 at the Poznań University of Medical Sciences^(27)^.

For Poland, the 1980s were a pivotal decade, marking the end of Soviet influence and the political transformation in 1989 that introduced democracy, pluralism, and social dialogue. In this new context, nurses focused on redesigning their practice and education at both basic and post-basic levels. One symbolic milestone for nursing was Poland’s adoption of the Bologna Process in 1999, an agreement signed by thirty European countries to ensure comparability in academic degree standards and quality^(27)^.

Freidson argues that the expansion and consolidation of a profession depend on professional systematization and the level of professionalism among its members. In the context of nursing, this is reflected in the establishment of educational centers, the regulation of the profession, and the development of a specialized body of knowledge^(1)^.

### Study limitations

Although the aim of this study is to map the global landscape, the analysis does not encompass all countries, as inclusion was based on the availability of published articles concerning each nation or region. This limitation was imposed by the quantity and scope of the available studies, resulting in the exclusion of some countries that were not represented in the reviewed literature.

### Contributions to the field of Nursing

The results of this scoping review contribute to the understanding of the institutionalization process of *stricto sensu* graduate education in the global context, highlighting its importance in the qualification of nurses.

## FINAL CONSIDERATIONS

This scoping review demonstrated that, over the decades, nursing has sought, through professionalization, to become a consultative profession, with its own scientific body of knowledge. Master’s and doctoral programs serve as pathways to expertise, thereby strengthening the professionalization process.

The articles analyzed in this review show that these discussions are particularly relevant in Brazil, contributing to the understanding and recognition of nursing within the global professional landscape. Nevertheless, it is important to highlight a significant gap in the scientific literature regarding the evolution of *stricto sensu* graduate programs in Nursing, resulting in a limited understanding of regional and temporal variations in the educational advancement of the profession.

## Data Availability

The research data are available within the article.
