# Supplementary material for: Sociological analysis of nurses’ qualification through stricto sensu graduate programs globally: scoping review
Source: Rev Bras Enferm. 2026 Jul 10;79:e20240560. doi: 10.1590/0034-7167-2024-0560 (PMC13363299; doi:10.1590/0034-7167-2024-0560)
Supplement: Translation

# Translation: Análise sociológica da qualificação dos enfermeiros pela pós-graduação *stricto sensu* globalmente: revisão de escopo

**DOI:** 10.1590/0034-7167-2024-0560pt

**Authors:** Bárbara Costa Moreira, Isabella Lara Maia de Carvalho, Rafaela Siqueira Costa Schreck, Camila Pureza Guimarães da Silva, Maria Itayra Coelho de Souza Padilha, Fernanda Batista Oliveira Santos

**Affiliations:** I Universidade Federal de Minas Gerais. Belo Horizonte, Minas Gerais, Brasil; II Universidade Federal do Rio de Janeiro. Rio de Janeiro, Rio de Janeiro, Brasil; III Universidade Federal de Santa Catarina. Florianópolis, Santa Catarina, Brasil

**Keywords:** Enfermagem, História da Enfermagem, Educação em Enfermagem de Pós-Graduação, Educação em Enfermagem, Pesquisa em Educação em Enfermagem.

## Abstract

**Objetivos:**

analisar o panorama das publicações científicas acerca do processo histórico da qualificação de enfermeiros por meio da Pós-Graduação *stricto sensu* em Enfermagem no cenário global, à luz da Sociologia das Profissões de Eliot Freidson.

**Métodos:**

revisão de Escopo, contemplando as bases de dados BVS, MEDLINE, Embase, Cochrane Library, Scopus e Web of Science. Utilizado descritores “História da Enfermagem”, “História e Educação de Pós-Graduação em Enfermagem” e seus correspondentes em inglês e espanhol, sob as recomendações do *Joanna Briggs Institute*, e o uso da diretriz *PRISMA Extension for Scoping Reviews*. Amostra final dos estudos totalizou vinte artigos.

**Resultados:**

os estudos discorreram sobre a história da Pós-Graduação *stricto sensu* em Enfermagem no Brasil, Chile, China, Polônia, Arábia Saudita, Jordânia, Gana e Estados Unidos da América.

**Considerações Finais:**

o estudo contribui para compreensão do processo de institucionalização da pós-graduação *stricto sensu* globalmente e da produção científica em enfermagem como instrumentos da profissionalização.

## INTRODUÇÃO

A evolução da enfermagem como profissão está intimamente relacionada ao processo de institucionalização do ensino, sobretudo por meio dos Programas de Pós-Graduação *Stricto Sensu* em Enfermagem (PPGENF). Mecanismos de profissionalização pautados nas transformações sociais e de saúde da população, os programas destacam a busca pela autonomia e reconhecimento da expertise dos enfermeiros, dinâmica interpretada pela aplicação da Sociologia das Profissões Eliot Freidson^(1,2)^.

O sociólogo Freidson argumenta que, para que uma ocupação seja reconhecida como uma profissão, ela deve possuir elementos essenciais: suas próprias instituições de ensino, um sistema de credenciamento, e uma regulação específica da prática e autonomia. Além disso, a profissão deve ter um conhecimento intelectual aprofundado e hierarquizado, o que demanda um elevado nível de qualificação profissional^(1)^.

Historicamente, a enfermagem moderna começou a se consolidar na Inglaterra na segunda metade do século XIX com Florence Nightingale, que estabeleceu a necessidade de uma formação formal e padronizada para enfermeiros. O modelo nightingaleano foi então adaptado e disseminado globalmente, principalmente pelos Estados Unidos da América (EUA), incorporando características capitalistas e tayloristas ao ensino de enfermagem. Essa adaptação visava aumentar a eficiência na educação dos profissionais da área^(3,4)^.

A institucionalização da Pós-Graduação em Enfermagem nos EUA norteou a prática de outros países, sendo este o primeiro país a ofertar cursos de mestrado e doutorado^(5-8)^. Desde 1924, o *Teacher’s College*, localizado em Columbia, Ohio, já ofertava programas de doutorado, sendo os seus egressos titulados Doutores em Educação (PhD). Tal titulação capacitava o enfermeiro para atuação na docência, apesar de não ser um programa específico para a área da enfermagem e saúde. O primeiro curso de doutorado inerente à enfermagem foi estabelecido em 1934 em Nova York, sendo este um importante marco para o processo de profissionalização^(9)^.

A expansão da pós-graduação *stricto sensu* em Enfermagem não se limitou ao contexto norte-americano. Diversos países, em especial na América Latina, passaram a investir na qualificação acadêmica de enfermeiros, impulsionando a construção de saberes próprios e o fortalecimento da profissão^(4)^. O Brasil, nesse cenário, assumiu papel de destaque, liderando iniciativas na formação de mestres e doutores, e contribuindo significativamente para a consolidação da Enfermagem como campo científico e profissional na região^(4,5)^.

## OBJETIVOS

Analisar o panorama das publicações científicas acerca do processo histórico da qualificação de enfermeiros por meio da pós-graduação *stricto sensu* em enfermagem no cenário global, à luz da Sociologia das Profissões de Eliot Freidson.

## MÉTODOS

### Tipo de estudo

O presente estudo é uma revisão de escopo em que se busca identificar os tipos de evidências disponíveis em um determinado campo de estudo, analisar lacunas de conhecimento, e identificar as principais características ou fatores relacionados a um conceito^(10)^. Nesse sentido, formulou-se a seguinte pergunta de pesquisa: “No cenário global, como ocorreu o processo histórico de qualificação dos enfermeiros por meio da institucionalização da pós-graduação *stricto sensu* em Enfermagem?”.

Para esta revisão de escopo foram utilizadas as recomendações do Joanna Briggs Institute (JBI), desenvolvidas a partir dos procedimentos de Arksey e O’Malley, e elaboradas usando a diretriz PRISMA-ScR (*Preferred Reporting Items for Systematic Reviews and Meta-Analyses extension for Scoping Reviews*), revisadas por duas avaliadoras, e com um protocolo de pesquisa com registro público na Plataforma *Open Science Framework* (https://osf.io/vm4bd/)^(11,12)^.

### Procedimentos Metodológicos

Para a elaboração deste estudo, foram percorridas cinco etapas: 1) Formulação da questão norteadora da pesquisa; 2) Definição dos objetivos da pesquisa, estabelecendo os critérios de inclusão e exclusão; 3) Criação da estratégia de busca e definição dos Descritores em Ciências da Saúde (DeCS); 4) Categorização dos estudos encontrados; 5) Interpretação e apresentação dos resultados.

### Coleta e Organização dos Dados

A coleta de dados ocorreu de janeiro a março de 2024 através das bases de dados LILACS e outras bases via Portal Regional da BVS, MEDLINE via PubMed e, via portal CAPES, a Embase, Cochrane Library, Scopus e *Web of Science*. Recuperaram-se estudos que continham pelo menos um dos termos dos conceitos História da Enfermagem e Pós-Graduação em Enfermagem. Foi realizada a identificação dos termos controlados (descritores DeCS/MeSH) e termos livres (palavras-chave), sendo eles História da Enfermagem e História e Educação de Pós-Graduação em Enfermagem, e seus correspondentes na língua inglesa e espanhola.

### Critérios de seleção de fontes

Os textos encontrados foram selecionados a partir da avaliação do título e do resumo, realizada por um revisor primário e um secundário, de forma independente, por meio do *software Rayyan* ^®^. Foram incluídos neste estudo artigos científicos, editoriais e notas de pesquisa em todos os idiomas. Não se definiu limite temporal para inclusão frente à necessidade de mapear as produções de forma extensiva e abrangente. Foram excluídos os textos não disponibilizados na íntegra e os que não atendiam ao critério de abordagem da Pós-Graduação *Stricto Sensu*. A elegibilidade contou com a abordagem acerca da Pós-Graduação *Stricto Sensu* no cenário global.

Os estudos foram pré-selecionados a partir da avaliação de títulos e resumos por um revisor primário e um secundário, de forma independente. Os casos de dúvida para seleção foram resolvidos em reunião de consenso entre os revisores. Os artigos pré-selecionados foram lidos na íntegra, com o objetivo de identificar a relevância para a pesquisa e, de forma mais precisa, os critérios de inclusão e exclusão. Nesta última etapa, extraíram-se os dados mais importantes para a análise.

### Análise dos Dados

A análise dos estudos foi realizada de forma descritiva, agrupando os artigos conforme os seguintes aspectos: contextos sócio-históricos, articulações institucionais e incentivos governamentais para a institucionalização das pós-graduações *stricto sensu* no cenário mundial. Posteriormente, procedeu-se à síntese narrativa das características metodológicas e temáticas elencadas dos estudos.

## RESULTADOS

A amostra final da presente revisão de escopo totalizou vinte estudos selecionados, com níveis de evidência V e VI^(13)^. O processo de busca e inclusão dos artigos está ilustrado no fluxograma (Figura 1) conforme recomendações do JBI, segundo lista adaptada do *Preferred Reporting Items for Systematic Reviews and Meta-Analyses Extension for Scoping Reviews* (PRISMA-Scr)^(10-12)^.

**Figura 1:** Diagrama do processo de busca e seleção de artigos, de acordo com adaptação do PRISMA-Scr

Os estudos abordaram o processo histórico da qualificação de enfermeiros por meio da pós-graduação *stricto sensu* no Brasil (n=13), Chile (n=1), China (n=2), Polônia (n=1), Arábia Saudita (n=2), Jordânia e Gana (n=1), e Estados Unidos da América (n=1). Considerando a proposição do surgimento da Pós-Graduação *Stricto Sensu* em Enfermagem no cenário global nesta amostragem, a Figura 2 esquematiza em ordem cronológica o princípio dos cursos de mestrado e/ou doutorado.

**Figura 2:** Linha do tempo do surgimento da pós-graduação *stricto sensu* em Enfermagem no mundo

A caracterização da produção científica acerca do processo histórico de qualificação da pós-graduação *stricto sensu* no cenário global está apresentada no (Quadro 1).

**Quadro 1:** Caracterização da produção científica acerca do processo histórico de qualificação da pós-graduação *stricto sensu* no cenário global

Código ID	País/Ano	Título	Delineamento	Nível de Evidência	Intervenções e Desfecho
PC1^(14)^	Brasil (2002)	A pós-graduação na escola de enfermagem de Ribeirão Preto - USP: evolução histórica e sua contribuição para o desenvolvimento da enfermagem	Análise quantitativa da produção da Pós-Graduação, e em bibliografias sobre o assunto.	Nível V	A Escola de Enfermagem de Ribeirão Preto da Universidade de São Paulo (EERP-USP) contribuiu para a formação de pesquisadores e foi designada em 1988 como Centro Colaborador da Organização Mundial da Saúde para o desenvolvimento da pesquisa em enfermagem. A EERP, com seu Programa de Pós-Graduação consolidado, foi referência em convênios com outras instituições para a formação de doutores. Firmaram-se convênios institucionais com a Universidade Federal da Bahia (UFBA, março de 1995), Universidade Federal da Paraíba (UFPA, março de 1995), Universidad de Concepción - Chile (UdeC, março de 1996) e Universidade Federal do Rio Grande do Norte (UFRN, março de 1997).
PC2^(7)^	Chile (2004)	Postgrado *Stricto Sensus* del Departamento de Enfermería de la Universidad de Concepción: Concepción, Chile	Análise documental.	Nível V	O curso de Enfermagem da Universidade de Concepción foi criado em 1947. Com mudanças epidemiológicas importantes no país na década de 2000, viu-se a necessidade de estabelecer níveis de pós-graduação *stricto sensu*. Neste estudo, não está posto o surgimento do curso de Mestrado em Enfermagem da universidade. Entretanto, o curso de Doutorado em Enfermagem começou suas atividades acadêmicas em março de 2004, apoiadas pela Universidade de São Paulo.
PC3^(15)^	Brasil (2005)	A trajetória histórica do Programa Interunidades de Doutoramento em Enfermagem e sua contribuição para a enfermagem brasileira	Análise documental.	Nível V	As Escolas de Enfermagem da Universidade de São Paulo (USP) dos campi de São Paulo e Ribeirão Preto tinham o desejo de criar um programa de doutoramento na área. Em 1979, as duas Escolas apresentaram à Câmara de Pós-Graduação da USP suas propostas para a criação do Programa. O parecer final foi de que não havia massa crítica para as referidas Unidades oferecerem isoladamente o doutoramento, recomendando a organização de um curso único envolvendo as duas escolas. Tal trabalho foi realizado pelas professoras das escolas Wanda de Aguiar Horta, Amália Correia de Carvalho, Evalda Cançado Arantes, Maria Aparecida Minzoni, Maria Helena Machado e Nilza Tereza Rotter Pelá. O Programa Interunidades de Doutoramento em Enfermagem foi aprovado pela Câmara de Pós-Graduação da USP em 1981. Instalava-se, então, o primeiro Doutorado em Enfermagem do Brasil e da América Latina. Em junho de 1986, o Programa foi credenciado pelo Conselho Federal de Educação (CFE), atual Conselho Nacional de Educação (CNE).
PC4^(16)^	Brasil (2005)	História da Pós-Graduação na Escola de Enfermagem da Universidade de São Paulo	Estudo histórico e qualitativo.	Nível VI	A necessidade da Pós-Graduação em Enfermagem foi legalmente reconhecida em 1949, pois o Decreto 27.426, ao regulamentar a Lei 775 de 1949, que dispunha sobre o ensino de enfermagem no país, previu no artigo 3º que “além dos dois cursos ordinários, podem (ou poderiam) ser criados outros de pós-graduação, destinados a ampliar conhecimentos especializados de enfermagem ou de administração”.
PC5^(9)^	Estados Unidos (2005)	PhD, DNSc, ND: the ABCs of nursing doctoral degrees	Estudo histórico e qualitativo.	Nível VI	A formação doutoral nos EUA tem suas raízes na Europa, sendo influenciada fortemente pela educação alemã. O primeiro doutorado ocorreu nos EUA em 1924 no *Teacher’s College*, na Universidade de Columbia (CU). Em 1934, a Universidade de Nova York (NYU) ofertou o primeiro Doutorado em Enfermagem. Em 1950, a Universidade de Pittsburgh realizou o primeiro Doutorado em Enfermagem com ênfase na prestação de cuidados em enfermagem. Na década de 1960 existiam quatro modalidades de doutoramento: EdD (Doutor em Educacão); DNSC (Doutor em Ciências da Enfermagem); ND (Doutor em Enfermagem) com pós-graduação clínica, sem foco na pesquisa; doutoramento genérico.
PC6^(17)^	China (2005)	*International collaboration for developing graduate education in China*	Estudo histórico e qualitativo.	Nível V	Em 1990, o Comitê de Educação de Pós-Graduação (COGNE) encaminhou quinze profissionais enfermeiros chineses aos EUA para aperfeiçoamento, a fim de atualizar o corpo docente com a formação de Mestres em Ciências em Enfermagem. Desses quinze profissionais, somente quatro retornaram à China após tal oportunidade. Em 1994 iniciou-se o Programa de Licenciatura de Ensino Superior (POHNED) na Universidade Médica de Xi’an (XMU), sendo o primeiro programa de pós-graduação de Enfermagem na China. Tal programa formou 84 enfermeiros mestres de 1994-2001.
PC7^(18)^	Brasil (2006)	Uma história de sucesso: 30 anos da Pós-Graduação em Enfermagem da UFSC	Pesquisa sócio-histórica e qualitativa.	Nível VI	Durante a década de 1950 e início dos anos 1960, houve grande ênfase para a formação de recursos humanos de nível superior no exterior, financiada por instituições de fomento tais como: Conselho Nacional de Desenvolvimento Científico e Tecnológico (CNPq), Coordenação de Aperfeiçoamento de Pessoal de Nível Superior (CAPES), Fundo do Desenvolvimento Técnico e Científico (FNDCT), Fundação de Amparo à Pesquisa do Estado de São Paulo (FAPESP), e instituições internacionais como a Fundação Rockfeller e a Ford. Vários fatores contribuíram para a criação de cursos de pós-graduação no Brasil, dentre estes a mobilização pela reforma universitária, o crescimento das matrículas no ensino superior, a expansão quantitativa dos institutos e departamentos, a necessidade de docentes mais qualificados, e o aumento decisivo sobre a demanda para a pós-graduação.
PC8^(19)^	Brasil (2010)	Contribuição da Escola de Enfermagem Anna Nery para a formação de mestres em enfermagem no Brasil: (1972-1982)	Estudo histórico-social e qualitativo.	Nível V	O governo brasileiro recorreu à ajuda internacional, firmando acordos entre o Ministério da Educação e Cultura (MEC) e a *United States Agency for International Development* (USAID) que tinham como propósito a reforma do ensino superior. Um dos principais pontos acordados era que a reforma do ensino deveria se adequar à necessidade da expansão econômica do país. No final da década de 60, o governo instituiu comissões para a elaboração de propostas para a reforma da educação brasileira. Ao final de 1967, o governo instituiu a Comissão Meira Mattos, que apresentou propostas de alcance imediato. Em 1968, o governo instituiu o Grupo de Trabalho da Reforma Universitária (GTRU), que apresentou propostas de maior alcance para a reforma do ensino superior, baseadas nas propostas dos acordos MEC/USAID. As lideranças da enfermagem no Brasil já reconheciam a importância da investigação científica para o desenvolvimento da profissão e para a construção do seu corpo de conhecimentos. Evento emblemático desta preocupação é o XVI Congresso Brasileiro de Enfermagem, promovido pela Associação Brasileira de Enfermagem (ABEn).
PC9^(20)^	Brasil (2010)	O desenvolvimento da Pós-Graduação *stricto sensu* em Enfermagem no Brasil: estudo exploratório	Pesquisa documental.	Nível VI	Uma das primeiras estratégias para a qualificação de profissionais de enfermagem para o ensino de pós-graduação *stricto sensu* foi a realização de concursos de livre docência e doutorado, que conferiam o título aos aprovados. Era necessário aos candidatos um *habitus* de pesquisador, pois deveriam defender uma tese ou utilizar um trabalho próprio realizado anteriormente para participar deste processo seletivo. Na Escola de Enfermagem Anna Nery (EEAN), os concursos de doutorado e livre docência ocorreram nos anos de 1968, 1975 e 1977. Assim, a partir da década de 1970, foram criados cursos de Mestrado e Doutorado em Enfermagem.
PC10^(21)^	China (2012)	*Graduate nursing education in China*.	Pesquisa histórica e qualitativa.	Nível V	Em 1978, a China aderiu a um modelo de Política Aberta ao perceber que o ensino superior em enfermagem em outros países era desenvolvido. Dessa forma, em 1983 criou-se a primeira Escola de Enfermagem na Universidade Médica de Tianjin (TMU). Os Programas de Mestrado na China começaram em 1992 na Universidade Médica de Pequim (PUMC), aumentando para cinco em 1998, trinta em 2003, 58 em 2007 e 65 em 2009. Na China existem dois tipos de Programas de Mestrado: um de pesquisa, e outro clínico. O primeiro Programa de Doutorado ocorreu em 2003 na Segunda Universidade Médica Militar (SMMU). Como desafio encontrado pelos pós-graduandos está a concepção de que a enfermagem é uma subdisciplina da medicina, sendo a pós-graduação influenciada e condicionada a ela.
PC 11^(22)^	Brasil (2012)	A Pós-Graduação em Enfermagem brasileira faz quarenta anos: avanços, desafios e necessidades de novos empreendimentos	Editorial.	Nível V	Os Programas de Pós-Graduação em Enfermagem têm formado novos pesquisadores globalmente, sendo esse movimento o que garantiu a expansão da Pós-Graduação em Enfermagem brasileira. Sua visibilidade vem sendo construída pela ampliação do número de cursos, consolidação da formação de recursos humanos qualificados, pesquisa, e produção científica. Essa consolidação se traduz nos 523 Grupos de Pesquisa cadastrados no Conselho Nacional de Desenvolvimento Científico e Tecnológico (CNPq), nas 165 bolsas de Pesquisador do CNPq, nos 5.194 artigos publicados no triênio 2007-2009 com visível melhora na qualificação dos periódicos, e a indexação de quatro periódicos brasileiros de Enfermagem na Web of Science em 2010 e, finalmente, com a conquista de quatro programas com patamares de excelência nacional e internacional.
PC12^(23)^	Brasil (2013)	Programa de Pós-Graduação em Enfermagem da Universidade Federal de Santa Maria: trajetória e resultados	Estudo quantitativo, descritivo, exploratório e documental.	Nível V	Na década de 1920, enfermeiras graduadas pela Escola de Enfermagem Ana Nery iniciaram cursos de Pós-Graduação nos Estados Unidos da América com bolsas de estudo. Consequentemente, começaram no sudeste os primeiros investimentos no aperfeiçoamento profissional em relação à pós-graduação, fator propulsor para a criação desses espaços de ensino em outras regiões do país. Contudo, observa-se que atualmente os recursos e financiamentos ainda se concentram em algumas regiões, configurando assimetrias no panorama da Pós-graduação em Enfermagem no Brasil.
PC13^(24)^	Brasil (2013)	Pós-Graduação *Stricto Sensu* em Enfermagem no Brasil: avanços e perspectivas	Estudo histórico social e qualitativo.	Nível V	A pós-graduação brasileira foi instituída pela Lei de Diretrizes e Bases da Educação Nacional em 1961 e aprovada em 1965. Surgiu em um contexto político de promoção do desenvolvimento econômico do país, pela necessidade de mão de obra especializada para preencher os novos empregos criados pelo desenvolvimento previsto, e pela necessidade de cientistas, pesquisadores e técnicos. A enfermagem foi instituída como área de conhecimento específico com representação no Conselho Técnico Científico (CTC) da Coordenação de Aperfeiçoamento de Pessoal de Nível Superior por meio da Resolução nº 1 de 1987. As Escolas de Enfermagem e a representação da área na Coordenação de Aperfeiçoamento de Pessoal de Nível Superior mobilizaram-se no sentido de fazer face às dificuldades enfrentadas, buscando formas alternativas para viabilizar a formação pós-graduada de recursos humanos qualificados. Outro fato importante a ser destacado foi a mudança na sistemática de avaliação dos cursos, caracterizada pelo aumento do rigor nos critérios avaliativos, periodicidade trienal, e qualificação por programa em uma escala de 1-7.
PC14^(25)^	Brasil (2015)	Pós-Graduação em Enfermagem na Universidade de Brasília: aspectos históricos de um processo de construção coletiva	Estudo histórico e qualitativo.	Nível V	Na década de 1990, o corpo docente dos programas de Pós-Graduação em Enfermagem era predominantemente de enfermeiros-doutores do quadro da própria instituição. Quando este não era o cenário, integravam-se aos programas docentes de outras áreas do conhecimento da própria instituição.
PC15^(26)^	Brasil (2015)	Curso de mestrado da Escola Anna Nery 1972-1975: singularidades da formação e desafios na implantação	Estudo histórico-social e qualitativo.	Nível V	Para a implantação do primeiro curso de Mestrado em Enfermagem na Escola de Enfermagem Anna Nery, o MEC credenciou para atuação na pós-graduação *stricto sensu* docentes que haviam feito cursos de pós-graduação no exterior ou em outras universidades públicas brasileiras. Para composição completa do corpo docente foram convidados professores de outras áreas, como educação e filosofia, que já possuíam o título de mestre ou credenciamento para lecionar em cursos de pós-graduação *stricto sensu*.
PC16^(27)^	Polônia (2018)	*Nursing education in Poland - The past and new development perspectives*.	Estudo histórico e qualitativo.	Nível V	O primeiro Programa de Mestrado em Enfermagem foi estabelecido em 1977 na Universidade de Ciências Médicas de Poznań. Entretanto, a transformação do sistema de enfermagem na Polônia, advinda da libertação da Polônia da influência soviética, concretizou-se em 1989, com a democratização do sistema político. Nesse contexto, os enfermeiros iniciaram atividades intensivas e multidimensionais destinadas a redesenhar a sua prática e a sua educação nos níveis básicos e pós-básicos. A partir de 1995, no Departamento de Enfermeiras e Parteiras do Ministério da Saúde em Varsóvia, iniciou-se um trabalho intensivo para definir as competências funcionais dos cuidados de enfermagem no âmbito do novo modelo educativo. O principal documento foi a “Estratégia Europeia da Organização Mundial da Saúde (OMS) para a educação de enfermeiros e parteiras” adotado em 1999. No mesmo ano, foi assinada a Declaração de Bolonha, entre 30 países europeus representativos, documento que ajudou a moldar a educação atual dos enfermeiros na Polônia. Após a assinatura da Declaração, foram introduzidas alterações significativas nos processos formativos em enfermagem na Polônia, como a introdução de estudos em três etapas: Bacharelado em Enfermagem, Mestrado em Enfermagem e Doutorado em Enfermagem.
PC17^(28)^	Arábia Saudita (2020)	*Nursing Education in Saudi Arabia: History and Development*	Revisão integrativa da literatura.	Nível V	O primeiro Programa de Mestrado em Enfermagem no Reino da Arábia Saudita foi desenvolvido em 1987 na *King Saud University* (KSU), somente para estudantes do sexo feminino. A partir de 2013, foi possibilitada a matrícula de estudantes do sexo masculino. O único Programa de Doutorado em Enfermagem foi introduzido em 2019.
PC18^(29)^	Arábia Saudita, Jordânia, Gana (2020)	*Development of nurse education in Saudi Arabia, Jordan and Ghana: From undergraduate to doctoral programs*	Revisão integrativa da literatura.	Nível V	Os primeiros relatos de Mestrado em Enfermagem na Arábia Saudita datam de 1988, tendo sido ofertado somente para mulheres até 2014. O primeiro Programa de Mestrado em Enfermagem se deu na *King Saud University*, assim como o primeiro Programa de Doutorado em Enfermagem, de 2019. Na Jordânia o mestrado se iniciou em 1986, sendo todo o corpo docente composto por profissionais estrangeiros dos EUA, Reino Unido e Suécia. O doutorado foi criado em 2005 na Universidade da Jordânia (JU). Em Gana, antes da independência em 1957, o país seguia o currículo de enfermagem estabelecido pelo Conselho Geral de Enfermagem da Inglaterra e País de Gales. Após a independência, Gana formou a Associação de Enfermagem Registrada para manter um registro de enfermeiros e, tal como no Reino Unido, a formação centrou-se na saúde curativa hospitalar. Nenhuma universidade oferecia um doutoramento em enfermagem, embora estivessem disponíveis Programas de Mestrado em Enfermagem. Os membros do corpo docente com doutorado foram educados na Europa, nos Estados Unidos da América, na África do Sul e na Ásia, tendo o restante dos docentes obtido doutoramentos em outros departamentos de universidades ganenses, como em saúde pública, administração de saúde e psicologia.
PC19^(5)^	Brasil (2021)	Programa de Pós-graduação em Enfermagem da UFSC: 45 anos de contribuição para a internacionalização da enfermagem brasileira	Estudo histórico social, documental e qualitativo.	Nível V	A enfermagem brasileira implantou o primeiro Programa de Pós-Graduação *stricto sensu* na Escola de Enfermagem Anna Nery, em 1972. Nessa mesma década, foram criados outros oito cursos de Mestrado em Enfermagem em vários estados brasileiros. A criação destes cursos visava suprir a necessidade de formação qualificada de docentes e pesquisadores para atender às demandas do ensino superior, acompanhar o desenvolvimento dos cursos de pós-graduação stricto sensu, e fortalecer a pesquisa por meio de grupos e laboratórios de pesquisa, bem como fomentar a criação de cursos de doutorado e, com isso, ganhar visibilidade internacional.
PC20^(4)^	Brasil (2021)	Historicidade da pós-graduação em enfermagem no Brasil: uma análise da sociologia das profissões	Revisão integrativa da literatura.	Nível VV	A institucionalização da pós-graduação brasileira se deu em um cenário de desenvolvimento econômico no período do regime militar como uma estratégia para gerar mão de obra qualificada e proporcionar o deslocamento do país para o campo da pesquisa. Considerando que os programas de pós-graduação surgiram no país com o intuito de capacitar profissionais para lecionar no ensino de nível superior, bem como desenvolver a ciência e a prática baseada em evidências, este fenômeno na enfermagem brasileira começou a tomar força a partir dos anos 1960 com o desenvolvimento das especializações *lato sensu*, consolidando-se com a aprovação do primeiro curso de Mestrado em Enfermagem na Escola de Enfermagem Anna Nery, em 1972, e o primeiro curso de Doutorado em Enfermagem na Escola de Enfermagem da Universidade de São Paulo em 1981.

## DISCUSSÃO

A presente revisão mapeou a literatura científica no que concerne ao processo histórico mundial de qualificação de enfermeiros por meio da Pós-Graduação *stricto sensu* em Enfermagem. Foram encontrados artigos com níveis de evidência V e VI - isso se deve à natureza retrospectiva e observacional desses estudos, que frequentemente dependem de fontes documentais, relatos subjetivos e registros históricos. Tais achados permitiram que as pesquisadoras deste estudo realizassem uma interpretação dos conceitos de Freidson para a dinâmica sociológica do processo de profissionalização da enfermagem^(1)^.

As profissões são influenciadas por fatores intrínsecos e extrínsecos, sendo eles contextos históricos, sociais, políticos e culturais^(4)^. Na enfermagem, o processo de profissionalização ocorre pela transição do cuidado empírico para o científico, ocasionando um saber próprio que combina práticas baseadas em evidências com um profundo conhecimento teórico metodizado^(30,31)^. Na concepção freidsoniana, a profissão se diferencia de uma ocupação ao exercer controle sobre o próprio trabalho e, para isso, deve dominar três aspectos: o conhecimento, o credencialismo, e a autonomia^(1)^.

Assim, a institucionalização da Pós-Graduação em Enfermagem é um marco fundamental no processo de profissionalização, uma vez que envolve a construção de um corpo de conhecimentos especializados e o controle profissional sobre a formação e a prática dentro de padrões técnicos, tecnológicos, científicos e éticos, que requerem uma sistematização da assistência a ser prestada^(32,33)^.

Observa-se que a maioria dos estudos encontrados se refere à história da institucionalização da Pós-Graduação *Stricto Sensu* em Enfermagem no Brasil (65%)^(4,15-26)^. O volume relevante na amostra de produções historiográficas denota o empenho dos pesquisadores brasileiros em documentar, por meio da história, o avanço da profissionalização da enfermagem no país, movimento importante para a edificação da memória da profissão. Ressalta-se, no entanto, que alguns estudos adotam abordagens descritivas ou analógicas da história, sem necessariamente se apoiarem nos pressupostos metodológicos da historiografia. A similaridade e possibilidade de análise no campo da profissão do enfermeiro vêm sendo discutidas no Brasil também por pesquisadores da história da enfermagem^(32,34-36)^.

A implementação da pós-graduação no Brasil ocorreu em um momento de mudanças políticas e econômicas importantes^(5)^. O país, sob regime militar, vivia um contexto de transformação, impulsionado por uma combinação de políticas econômicas, industriais e tecnológicas que promoveram um crescimento econômico significativo. O avanço industrial provocou a necessidade de um mercado profissional mais qualificado, levando à eclosão de movimentos estudantis que exigiam maior acesso à educação^(4,5,25)^. Neste contexto, em 1968 ocorreu a Reforma Universitária, cujo objetivo era melhorar as condições das universidades, fortalecer a formação de docentes, instituir a pesquisa acadêmica, e aumentar a visibilidade dos cursos superiores no Brasil^(4,26)^.

Desta forma, em 1972 surge o primeiro curso de Mestrado em Enfermagem do país, na Escola de Enfermagem Anna Nery (EEAN), primeira escola de enfermagem do Brasil nos moldes do padrão nightingaleano^(26)^. Este modelo de formação, criado por Florence Nightingale em 1860, tornou-se padrão para as demais escolas de enfermagem fundadas posteriormente, com a instituição da prática de ensino metódico. A enfermagem, assim, passa de uma atividade empírica, desvinculada do saber especializado, para uma atividade disciplinadora e científica, constituindo-se como uma prática social institucionalizada e específica para atender às necessidades hospitalares^(37)^.

O primeiro curso de Doutorado em Enfermagem do Brasil e da América Latina foi criado em 1981 na Escola de Enfermagem da Universidade de São Paulo (EEUSP)^(15)^. Tal fato demonstra o protagonismo e liderança da enfermagem brasileira na consolidação de uma profissão pautada na produção de conhecimento esotérico. Freidson expõe que o conhecimento esotérico, denominado expertise, é um componente essencial para a autoridade técnica e teórica de uma profissão, pois baliza a autonomia através da construção de saberes específicos em seus centros educacionais^(1)^.

A nível global, os Estados Unidos da América (EUA) desempenharam um papel crucial na profissionalização da enfermagem. Diversos estudos demonstram o país como referência e apoio expressivo para o desenvolvimento da profissionalização da enfermagem no restante do mundo^(4,17,19,29)^. O modelo anglo-americano, baseado nos preceitos da enfermagem moderna e difundido internacionalmente pelos EUA, é uma evolução da prática de enfermagem que enfatiza a autonomia e o papel independente do enfermeiro, e se propagou como influência para a profissionalização da produção científica em enfermagem^(38)^.

Carregal observa que, enquanto a enfermagem norte-americana ofertava cursos de especialização *stricto sensu* e *lato sensu* em 1924, o Brasil estava construindo suas primeiras escolas de enfermagem baseadas no paradigma nightingaleano^(4)^. Ademais, os Estados Unidos da América contribuíram para a qualificação do corpo docente brasileiro, viabilizando, além da instituição do ensino superior em enfermagem no país, o desenvolvimento dos programas de pós-graduação^(38)^.

A China foi um dos países influenciados pelos EUA na profissionalização da enfermagem^(17)^. A primeira escola de enfermagem foi instituída em 1920, porém, em um contexto caracterizado por um controle estatal rígido, com ênfase na uniformidade ideológica em detrimento do desenvolvimento profissional e educacional. Neste período, a profissão não era compreendida como essencial no país, resultando no fechamento de vários centros de formação. Devido a questões socioeconômicas e políticas, em 1978, e com a adoção de um governo de abertura, os chefes de estado reconheceram a importância de desenvolver o ensino superior em enfermagem para acompanhar o progresso global e, em 1983, foi criada a Escola de Enfermagem da Universidade Médica de Tianjin (TJMES)^(21)^.

Para atender à demanda por profissionais qualificados, a China estabeleceu convênios e colaborações externas para aprimorar a educação em enfermagem. Sob a análise freidsoniana, esses movimentos corroboram o reconhecimento da enfermagem enquanto profissão autônoma, posto que a autonomia advém de um corpo especializado de conhecimentos adquiridos por meio de credenciais constituídas pela institucionalização de escolas e universidades^(2)^. O apoio estrangeiro introduziu padrões internacionais de ensino e prática de enfermagem no país, e o governo criou o Comitê de Educação de Pós-Graduação (COGNE) para enviar aos Estados Unidos enfermeiros chineses visando à estruturação de um corpo docente habilitado. De quinze profissionais, somente quatro retornaram à China após tal oportunidade^(17)^.

Com a imigração de capital humano, crescimento contínuo da profissão, e necessidade progressiva de mão de obra capacitada, em 1992 instituiu-se o primeiro Mestrado em Enfermagem na China, na Faculdade de Medicina da Universidade de Pequim (PUMC). O doutoramento foi estabelecido em 2003, na Segunda Universidade Médica Militar (SMMU)^(21)^. Desta forma, observa-se na China a construção da enfermagem como ciência e profissão por meio da constituição de campo de conhecimento próprio para subsidiar a prática profissional.

Assim como nos demais países, o Chile passou por reconfigurações econômicas e sociais, resultando em reformas educacionais e sanitárias para a melhora da qualidade dos cuidados de saúde. Nesta amostragem, não está posto o surgimento do primeiro Programa de Mestrado em Enfermagem no país. Por outro lado, estudos apontam que, sob a influência de outros países, houve uma iniciativa para formar uma massa crítica de acadêmicos em nível de doutoramento, com o objetivo de estabelecer o primeiro Programa de Doutorado em Enfermagem no Chile, na Universidade de Concepción (UdeC)^(7)^.

Tal programa teve forte influência da Universidade de São Paulo Campus Ribeirão Preto, que estabeleceu um convênio com a Universidade de Concepción, financiado pela Fundação Kellogg, organização norte-americana. Em março de 1996, quatro estudantes chilenas foram enviadas à USP para participarem do programa. Após concluírem o doutorado, elas retornaram ao Chile e ocuparam cargos acadêmicos importantes. Com essas credenciais, elas lideraram a criação do primeiro Programa de Doutorado em Enfermagem no Chile, que foi aprovado em 2003^(7)^.

Países africanos e do Oriente Médio como a Arábia Saudita, Gana e Jordânia também foram influenciados pelos Estados Unidos. A pós-graduação em enfermagem na Arábia Saudita surgiu em um contexto de desenvolvimento educacional e de modernização do sistema de saúde do país, que passou por uma série de transformações socioeconômicas importantes desde a descoberta de petróleo, na década de 1930. Inicialmente, o governo focava na importação de conhecimento estrangeiro e na formação de enfermeiros de nível básico. No entanto, impulsionado pela necessidade de formar profissionais aptos para atenderem à carência do sistema de saúde saudita, em 1988 foi criado o primeiro curso de Mestrado em Enfermagem na *King Saud University* (KSU), sendo ofertado apenas para mulheres até 2014. O Doutorado em Enfermagem foi introduzido posteriormente em 2019, na mesma universidade^(28,29)^.

Assim como nos demais países desta revisão, o sistema de saúde jordaniano foi influenciado pelo modelo anglo-americano à medida que o país procurava modernizar suas práticas de enfermagem e aumentar a qualificação de seus profissionais de saúde. As universidades jordanianas, em colaboração com instituições internacionais, estabeleceram programas de Pós-Graduação em Enfermagem que incorporaram padrões internacionais. Na Jordânia, o curso de mestrado iniciou-se em 1986, sendo todo o corpo docente composto por profissionais estrangeiros dos Estados Unidos da América, Reino Unido e Suécia. O doutorado foi instituído em 2005 na Universidade da Jordânia (JU)^(29)^.

Historicamente, assim como em outros países africanos, em Gana existem desafios consideráveis em termos de infraestrutura de saúde, recursos humanos qualificados, e acesso a cuidados de saúde adequados. A necessidade de melhorar a condição dos serviços do sistema de saúde em desenvolvimento impeliu o governo a colaborar com instituições internacionais para estabelecer programas de pós-graduação. O primeiro mestrado foi instituído em 2007, na Escola de Enfermagem e Ciências da Saúde da Universidade de Gana. Inicialmente, o corpo docente era formado na Europa, nos Estados Unidos da América, na África do Sul, e na Ásia. Não há estudos nesta amostragem acerca de cursos de Doutorado em Enfermagem em Gana; no entanto, alguns enfermeiros obtiveram doutoramento em outros departamentos de universidades ganenses, como em saúde pública, administração de saúde, e psicologia^(29)^.

Após a Segunda Guerra Mundial, a Europa atravessou mudanças sociais, econômicas e culturais significativas que influenciaram a saúde da sociedade europeia. A Polônia, país do Leste Europeu que foi um dos países ocupados militarmente pela Alemanha, no pós-guerra vivenciou muitas epidemias, incluindo de doenças infecciosas, além do desmantelamento de conquistas pré-guerra na cultura, ciência e educação polacas^(27)^.

Neste período pós-guerra, devido ao contexto sociopolítico e à necessidade de profissionais de enfermagem, a Polônia contava com profissionais de formação limitada com cursos secundários de seis meses para suprir as demandas dos hospitais. Concomitantemente, algumas escolas de enfermagem anteriores à guerra foram restabelecidas, bem como a Associação Polaca de Enfermagem, responsável pela criação da Faculdade de Enfermagem na Academia Médica de Lublin em 1969, com um curso de três anos. Esta iniciativa deu origem a outras escolas de enfermagem, entre elas Poznań, Cracóvia, Katowice e Wrocław, onde os enfermeiros foram formados em nível de pós-graduação para receberem o título de Mestre em Enfermagem. O primeiro programa de Mestrado em Enfermagem foi estabelecido em 1977, na Universidade de Ciências Médicas de Poznań^(27)^.

Para a Polônia, a década de 1980 foi de extrema importância, pois marca a libertação da influência soviética e da mudança do sistema político em 1989, introduzindo no país a democracia, o pluralismo e o diálogo social. Nesse cenário, os enfermeiros debruçaram-se em redesenhar a sua prática e a sua educação nos níveis básico e pós-básico, e um dos momentos simbólicos para a enfermagem foi a adesão ao Processo de Bolonha em 1999, um acordo firmado entre trinta países europeus para assegurar a comparabilidade nos padrões e na qualidade dos diplomas acadêmicos^(27)^.

Freidson argumenta que a expansão e consolidação de uma profissão dependem da sistematização profissional e do nível de profissionalismo de seus membros. No contexto da enfermagem, isso se reflete na consolidação de centros educacionais, na regulamentação da profissão e na construção de um corpo de conhecimento especializado^(1)^.

### Limitações do estudo

Embora o objetivo do estudo seja mapear o cenário global, a análise não abrange todos os países, uma vez que a inclusão se baseou na disponibilidade de artigos publicados sobre cada nação ou região. Esse limite foi imposto pela quantidade e cobertura dos estudos disponíveis, resultando na exclusão de alguns países que não estavam representados na literatura revisada.

### Contribuições para a área da enfermagem

Os resultados desta revisão de escopo contribuem para a compreensão do processo de institucionalização da pós-graduação *stricto sensu* no cenário global, evidenciando sua importância na qualificação dos enfermeiros.

## CONSIDERAÇÕES FINAIS

Esta revisão de escopo demonstrou que, ao longo das décadas, a enfermagem vem buscando, por meio da profissionalização, tornar-se uma profissão denominada de consulta, com seu saber próprio e científico. O mestrado e o doutorado são caminhos para a expertise, fortalecendo, dessa forma, o processo de profissionalização.

Os artigos analisados nesta revisão demonstram que essas discussões são particularmente relevantes no Brasil, contribuindo para a compreensão e valorização da enfermagem no cenário profissional global. Apesar disso, ressalta-se a existência de uma lacuna expressiva na literatura científica acerca da evolução dos programas de Pós-Graduação *Stricto Sensu* em Enfermagem, resultando em uma compreensão limitada das variações regionais e temporais no avanço educacional da profissão.

## Data Availability

Os dados de pesquisa estão disponíveis no corpo do artigo.
